# Free to err? Conceptualising personal autonomy in the postpandemic welfare state

**DOI:** 10.1080/11287462.2025.2518800

**Published:** 2025-06-18

**Authors:** Marc Sørensen, Ezio Di Nucci, Karsten Juhl Jørgensen, Gorm Greisen

**Affiliations:** a Department of Cardiothoracic Anaesthesiology, Copenhagen University Hospital–Rigshospitalet, Copenhagen, Denmark; b Department of Public Health, Faculty of Health and Medical Sciences, University of Copenhagen, Copenhagen, Denmark; c Centre for Evidence-Based Medicine Odense and Cochrane Denmark (CEBMO), Department of Clinical Research, Faculty of Health and Medical Sciences, University of Southern Denmark, Denmark; d Department of Neonatology, Copenhagen University Hospital–Rigshospitalet, Copenhagen, Denmark; e Department of Clinical Medicine, Faculty of Health and Medical Sciences, University of Copenhagen, Copenhagen, Denmark

**Keywords:** Personal autonomy, paternalism, COVID-19, coercion, ethics, vaccination, decision-making

## Abstract

In the public healthcare system, personal autonomy has rightly become a key element of health politics. Nevertheless, conflicts can arise when the interests of a caring welfare state collide with the decisions of those in its care. In such cases, the concept of autonomy as a fundamental ethical principle can cause harm, if solely interpreted as freedom from interference by the public sector, devoid of demands on personal responsibility. Based on the example of vaccine hesitancy during COVID-19, we propose that resolving these conflicts should integrate two divergent basic tenets of autonomy, as developed over time, and recently applied disjunctively to the pandemic by ethicists, so that the principle can be consistently operationalised as a function of an open but also binding argument within society. This may have implications far beyond SARS-CoV-2. We touch on philosophical grounds where the assertion of axiomatic moral rights is replaced by an epistemological framing of the deliberation process as a ubiquitous and not merely representative argumentative act, while validity claims are individually redeemed in a dialogical balancing of reasons and objections. Recognising this as the humanistic core of healthcare calls for an expansion of the state’s communicative obligations but also implies proportionate paternalism.

## Introduction

During the second year of the coronavirus disease 2019 (COVID-19) pandemic,^1^ one of the authors noticed a marked change in the characteristics of patients (*n* = 18) admitted to the Copenhagen Centre for Extracorporeal Membrane Oxygenation (ECMO) due to pulmonary infection with the severe acute respiratory syndrome coronavirus-2 (SARS-CoV-2). By that time, most Danes^2^ had been vaccinated against COVID-19 and although the early variants were gradually being overtaken by the more infectious Delta, all responded to the novel vaccine. The main mRNA-vaccine in use (Comirnaty®, Pfizer/BioNTech) had proven to be highly effective and safe.^3^ As a result, the centre fortunately had fewer patients on ECMO than previously; but those were younger (mean age 46 years), had fewer comorbidities, longer hospital stays and unaltered mortality rates. Eleven patients died during treatment. Although it might be reasonable to assume they had been vaccinated, none of the patients put on ECMO in this period had been.

The potentially avoidable suffering and death of these young and otherwise healthy individuals, as well as the difficulty their care occasionally posed in meeting other patients’ needs, prompted staff to consider different explanations for why they had not been vaccinated and how these patients might have been. The doctors doubted that more information would have sufficed, believing that the extensive official education campaigns across the country meant that people were generally well-informed of the authorities’ recommendations. This finding coincided with reports on the reasons for vaccine hesitancy or refusal in other contexts^4^ (Kärki, 2022). Correspondingly, the vaccination rate in Denmark was consistently high throughout 2021. However, the broad and ongoing discourse on how to address this opposition focuses predominantly on the population impact and neglects the notable difference minor changes in the overall vaccination compliance could have made to everyone whose life was at risk. Indeed, this discourse is neither limited to COVID-19 nor vaccination policy but is constantly repeated in every new and different public healthcare relationship between the individual and society (Kärki, 2022). SARS-CoV-2 has challenged the global community socially, logistically, economically and in terms of health and welfare, at every level. But, beyond that, some patients may have been treated for diagnoses other than COVID-19 and yet have contributed to their illness through informed, albeit risky, lifestyles.^5^ Thus, the social and individual costs of various actions and circumstances raise the fundamental question of whether we (society) should restrict (individual) autonomy, one of the prominent principles of medical ethics (Gómez-Virseda & Usanos, 2021) if it leads to choices with potentially fatal consequences.

Now, has COVID-19 provided new insight into the well-known contraposition of personal autonomy and institutional paternalism in public healthcare (Dunne & Spain, 2023)? To answer this, we will:

1. outline the concept of personal autonomy underlying a public healthcare system such as the Danish,
2. relate it to recent contributions on COVID-19 and finally,
3. suggest that from a philosophical point of view, personal autonomy in healthcare should be understood and treated as assuming personal responsibility.

## Personal autonomy before COVID-19

Traditionally, solutions to the question surrounding the ethically best possible distribution of healthcare^6^ on the one hand, contra their associated costs on the other, have varied depending on whether the perspective is utilitarian, egalitarian, communitarian, or libertarian or if it originates from the attempt to reconcile several of these (Rawls). Although patient autonomy gained greater importance in the 2000s through the concept of shared decision-making and self-government, in public healthcare systems such as the Danish, equal individual access to healthcare usually takes relative precedence over the value of collective utility and personal freedom. The principle of equity is meant to ensure that personal circumstances, including social factors,^7^ do not determine access to healthcare. Allocating resources to those with the greatest need rather than the more resourceful sections of society, stops health inequality from worsening. Neither does a patient’s behaviour usually affect access to healthcare, nor the disposition to pay (extra) for it. The Danish healthcare system is largely tax-funded, and taxes are progressive, based on income. In the case of organ transplantation, for instance, which has been compared to ECMO in cost-effectiveness analyses, neither the willingness to donate nor prior smoking or alcohol issues have any influence on decisions regarding graft allocation, provided contemporaneous and future abstinence can be substantiated. Similarly, vaccination willingness was not deemed a prerequisite for ECMO during the COVID-19 pandemic either.

However, as previously mentioned, patient autonomy enjoys broad political support in Denmark. Self-government and self-determination have been central to Danish health legislation since their introduction as a legal right in the 1998 Patients’ Rights Act (Folketinget, 1997). Autonomy in this context is understood as every person being entitled to live according to his or her own beliefs and values, and that the individual is best placed to make decisions regarding important personal matters, including those situations where others might choose differently. It also recognises that many, if not all, decisions in healthcare, even those on the magnitude of a pandemic threat, the safety and effectiveness of a vaccine, as well as the cost/benefit ratio of the use of compulsion (Savulescu, 2021), are essentially value judgments, and that the value of a specific health intervention in terms of the benefits or harm to an individual may differ substantially from person to person. According to António Barbosa da Silva, this view is based on post-Kantian humanism, which dominates the conception of personal autonomy in Western Europe and plays an important role in patient rights legislation nowadays (da Silva, 2009), as seen in the widespread use of informed consent requirements (Toebes et al., 2022). Now enshrined in the Danish Health Act, these include the right to physical and personal data self-determination, access to health-related information and to choose freely between treatments if alternatives are provided, but do not include the right to demand treatments that are not. Accordingly, organ donation for transplantation must be officially approved during the donor’s lifetime or by the bereaved.

Despite these undoubtedly important legal landmarks, patient autonomy in healthcare may still be conceptually underdeveloped. Some authors regard it as being clinically dominated by a default, rather simplistic Cartesian variant of principlism; promoting an abstraction of the concept that refers to the utopian, self-transparent and self-interested, competent individual at a distinct and ahistorical point in time, atomistically isolated from any sociocultural interdependencies (Gómez-Virseda & Usanos, 2021), lacking the value of personal coherence, intentional content, self-reflection and self-control (Dive & Newson, 2018). Importantly, it normally also comes with a restriction: it can only be exercised if doing so does not cause harm to others (Mill’s so-called “harm principle”). This is the fundamental principle that allows, e.g. speed limits and seat belt requirements, gun control, and laws against physical violence as part of parenting and in other settings to be implemented by society although each may substantially limit personal autonomy.

Hence, one can argue that noncompliance with vaccination policies would also harm other citizens, either directly through contagion or indirectly through opportunity costs caused by resources allocated to the treatment of infected vaccine opponents, and that requirements to get vaccinated are justified and not different from other societal requirements to limit spread, which represent societally imposed limits to individual autonomy. In fact, this is how restrictions, and their justification were predominantly theorised during the pandemic (Toebes et al., 2022).

## Personal autonomy during COVID-19

If the concept of autonomy was controversial before COVID-19, the pandemic with its extraordinary requirements for patient isolation, social distancing, rationing of limited medical resources, stigmatisation, personal tracking, etc., has turbocharged the debate: the individualistic concept of autonomy has been criticised as being inappropriate given the unprecedented political challenges (Pedersen et al., 2025). David Ian Jeffrey, for instance, claims that healthcare workers struggled ethically with a basic shift from the individual-oriented to this public-centered approach. There has been notable tension between the authorities’ power to enforce mandatory measures and their duty to limit any resulting harm to individuals and communities. Trust and solidarity among the population have been put under pressure (Dive & Newson, 2018).

During the COVID-19 pandemic the Danish Health Authority, therefore, chose to strongly recommend vaccination, supported by the requirement of negative test results for access to public events, while at the same time also respecting the individual’s freedom to refuse. The concern was that introducing mandatory vaccination would lead to polarisation, erode trust in health authorities and health advice in a broader sense, potentially resulting in lower vaccination rates. In contrast and given that different policies may be called for in different contexts, governments in, e.g. Germany and Austria voted for mandatory vaccination as a prerequisite for the freedom of movement, although the implementation of these directives has varied. Similarly, Julian Savulescu, Jonathan Pugh and Dominic Wilkinson advocate the use of proportionate compulsion based on the vaccine’s effectiveness, safety and cost/benefit profile as well as the extent of the pandemic threat (Savulescu, 2021; Savulescu et al., 2021). While Susan Pennings and Xavier Symons in their response argue against this, noting the significant potential for lower vaccination rates due to a loss of trust in the authorities among the vaccine hesitant (Pennings & Symons, 2021), Michael Kowalik refutes the use of coercion on the grounds that it would conflict with the most basic ethical principle of physical integrity and human agency (Kowalik, 2022). Like Savulescu et al. and Pennings et al. he predominantly focuses on the limited role altruism and justice play in the relationship between the individual and society. Even in the case of significant public benefit and with little risk for the individual, there would still be no moral obligation to be vaccinated. At any rate, personal autonomy and its scope seem to have been inconsistently discussed during COVID-19, either as empowering the individual to satisfy his or her needs or as a moral bulwark against society's growing demands under the extraordinary conditions of the pandemic.

## Personal autonomy in future

It seems intuitive to assume that individuals would comply with the needs of society if vaccination were the only way to avert a disastrous event, such as an Ebola outbreak with a case fatality rate of 50 percent and no available cure. Authors who, like Kowalik, are opposed to mandatory vaccination *in principle* should apply such distinctive scenarios to their argument, as they may be basing their concerns on the premise of the relatively milder impact of COVID-19. Moreover, they neglect five circumstances that are of particular importance:

1. that not only the wellbeing of others is at risk but also the individual’s own, as the above example illustrates,
2. that we usually do not know whether this risk has been reasonably assessed by the individual who chooses to refuse vaccination,
3. that the opportunity costs associated with the individual’s preventable critical illness can be high,
4. that the disease’s progress, in the example given, could not be predicted, as it occurred as a rare event in otherwise healthy adults, and finally
5. that mandatory vaccination does not equal physical coercion but can be gradually and proportionately adapted on a sliding scale from requesting understanding and reason, to persuasion followed by mild, then moderate, then severe limitations of civil rights (as has been suggested by Savulescu).

To integrate all these aspects, the public healthcare system needs an ethical framework based on the reciprocal demands of society and its members, which is more elaborate than what has been presented thus far. At the very least, ethical theory should clarify how the magnitude of the pandemic threat could make a relevant normative difference. In particular, the tenets of autonomy as a positive goal of healthcare on the one hand, and as the individual’s negative moral right to reject public-sector interference on the other, could easily complement each other more constructively, as valuable suggestions supporting both strands have already been forthcoming (Dive & Newson, 2018; Dunne & Spain, 2023; Jeffrey, 2020; Kärki, 2022). In his critique of Beauchamp and Childress as well as Savulescu, Quentin Genuis describes the historical development of the concept of autonomy as a yet nonovercome dichotomy of Kantian and Millian ethics and suggests that the origin of this gap could be its political interpretation and the corresponding attempts to redirect power in the relationship between doctors and patients (from doctors to patients in the twentieth century, and since back) (Genuis, 2021). While Kant invented the concept of autonomy as a positive moral obligation for the individual to act in concordance with the categorical imperative, Mill developed it as a negative political right to freedom from interference by others. None of them understood their concept as a contribution to what we now call bioethics, but both at least explicitly and discrepantly used the example of suicide. For Kant the act of (allowing one) killing (him – or herself), and self harming in a broader sense, is not universalizable and hence not ethical. For Mill it can be an expression of free will and should then be respected. Genuis shows how Kant and Mill mark a spectrum of historical conceptions of autonomy and also signify a gap in relation to modern physician-patient relationship. However, instead of referring to the somewhat vague existentialist interdependence in this relationship, as suggested by Genuis, we propose these agents’ communicative encounter itself as being the “integrator and uncoverer” of concept and misconception. The theoretical integration of patients’ active empowerment and physicians’ respect for their self-directedness with its limits reaches well beyond the discussion raised by COVID-19. It could offer solutions to the yet unresolved conflicts between the individual and society at large, e.g. the issues over the shortage of organs for transplantation, the definition of medical futility, the acceptability of physician-assisted dying, and the rationing of healthcare. But it could, and perhaps should, also expand and clarify the epistemological prerequisites of these conflicts, in as much as the meaning of the term “misconception” needs exploration (Sørensen & Andersen, 2019). It may be operant not only between the individual and society but also within the individuals’ mindset.

We suggest that the source of many of these controversies may lie in an inappropriate framing of the individual’s commitment to society in terms of moral rights and duties, where the concept of rights, and herein the ethical principle of autonomy (as Kowalik stresses) may prove unsuited to providing a proper ethical foundation in situations such as those described. It might prove more constructive to frame the process as *a statement of descriptive or normative facts* – and thus, as subject to justification. Since we never affirm an individual patient’s right to potentially harmful treatment unless that person has been sufficiently informed, we could assume that the right to vaccine refusal is also inherently linked to reasons put forward through considered argument. This could apply not only to a given benefit/harm scenario for third parties, but also to the expected benefit/harm for the individual him – or herself. If citizens make decisions for good reasons that endanger their lives or the lives of others, we may sanction this, but if they do so without offering any reason, or with reasons that cannot be shared then we should not grant it out of a blind respect for their axiomatic rights. Instead, we should engage them in an argumentative and retroflexive dialogue and, if this is not (yet) possible for some reason, also prevent them from making these decisions. We argue that this approach both constitutes and integrates respect for the individual as a competent citizen in the welfare state with an ethos of mutual help and protection. According to this understanding, misconception under ideal communicative conditions equals the residuum of open, and thus, legitimate, objections to the presented proposition (Wohlrapp, 2014).

Kaisa Kärki provides a valuable holistic approach to addressing vaccine refusal as an exit strategy from individual commitment to society, and suggests engaging in just such a serious, explorative and respectful dialogue (Kärki, 2022). In contrast, vaccine refusers during the COVID-19 pandemic have not been confronted often enough by exploring and challenging their reasons. Communication has mainly been a monologue. We know very little of the individual’s motives, and the Danish primary healthcare sector has not been sufficiently involved in this task. Thus, instead of the individual’s commitment to society we demand his or her commitment to his or her own inferences, which also implies self-responsibility. Framing personal autonomy as a question of good arguments rather than the assertion of moral rights and their corresponding obligations offers several additional advantages:

1. it explains how compulsion can be justified (when the individual vaccine refuser’s arguments are unknown but can be assumed to be unsound or invalid or when refuted motives are not dismissed),
2. it allows concurrent dialogue and coercion as long as (1.) remains uncontradicted,
3. it ensures the representation of those concerned who, for one reason or another, cannot take part in the deliberation,
4. it addresses the fundamental limits of knowledge and understanding, as well as the value of the individuals’ complementary trust in society (Conradsen et al., 2024) and
5. it, in turn, provides a more solid foundation for a flexible and pragmatic way of developing public healthcare services.

COVID-19 has made it clear that autonomy and responsibility are two sides of the same coin for the authorities as well as for citizens, whereas this relationship has traditionally been underexplored in classic bioethics. A notable exception is a recently published article by Jin Park and Ben Davies (Park & Davies, 2023). Their literature review on the question of whether refusal to vaccinate against COVID-19 should be sanctioned by rationing scarce Covid care is comprehensive but, too, misses the opportunity to stress that validity claims (and thus the reasons for or against vaccination) are necessarily dialectical and, due to their “openness”, contestable in principle. Since the attribution of the descriptively or normatively right or wrong presupposes transjubjectivity (Wohlrapp, 2014), the question of how to deal with vaccine refusers (especially in the event of a healthcare shortage) must include a bilateral dialogue about the refusal and its consequences in advance. None of the cases presented by Park and Davies should be resolved either by these cases’ protagonists alone or without them. The individual should always be directly involved in personal health matters; not only for legal reasons but also because of the unique perspective and argument he or she can provide in a decision-making process that ultimately has their best interests at heart. The argumentative involvement of individuals should always be preferred to their deliberative representation. Nevertheless, the individual's assessment of a given situation, their appraisal of their personal goals set against those of society, can also prove to be erroneous. The more complex the circumstances, the more likely it is that this will happen (Kärki, 2022). To err is not a moral prerogative.

## Discussion

In the past five years, COVID-19 has clearly affected and challenged the autonomy of most citizens in welfare states. The conflicts that arose have been widely discussed in both scientific and public fora; on the one hand in relation to mandatory measures such as vaccination, and on the other in relation to paying heed to what the patient wants in difficult conditions such as isolation and quarantine. These dual, and at times competing discourses have been conducted independently of each other, and to the authors of this paper this suggests that personal autonomy as a fundamental concept for publicly regulated healthcare systems has not been sufficiently developed – and not just in the context of COVID-19.

An initial step towards solving these conflicts might be to link the two discourses, protection from public-sector interference and individual empowerment, to facilitate responsible decision-making by the individual. To the extent that the healthcare system supports this, it should be considered ethically acceptable even if it acts paternalistically (Sjöstrand et al., 2013). The next step could be to question the longitudinal framing of such conflicts as inherently a matter of axiomatic moral rights and instead framing them as an argumentative exploration of validity claims. Regardless of its feasibility, this approach would have the advantage of presupposing what both steps would amount to: an unbiased dialogue between the individual and society that encourages both parties to acknowledge each other's arguments. But it would also mean a substantial reconsideration of the received view of autonomy. On this view, freedom from persuasion (or coercion), the ability to understand relevant information and to reason through the implications are necessary and sufficient conditions for choosing autonomously (Graber, 2021). Contemporary bioethicists in the tradition of Kant and Mill typically agree on this. For them, refusing to engage in the argument would indeed be a permissible exercise of autonomy. The example in this paper, however, shows that respecting private, but possibly erroneous idiosyncrasies in the name of autonomy can be fatal for the individual and have major opportunity costs for society. There is much evidence to suggest that a patient’s initial decision not to be vaccinated against COVID-19 may not only have been inadequately informed and thought through but also that many individuals and their relatives would probably have regretted it in retrospect. Rather than being a matter of the improbable, yet unlucky, result of a conscious calculation based on well-informed risk-estimates, decisions may be made without constructive opposition offered in the patient’s best interest,^8^ although concerns that mandatory vaccination without any explanatory discourse would polarise public opinion, reduce vaccination rates and cause even higher opportunity costs would seem to be warranted. Given the complexity of the pandemic challenges, a solid basis for refusal cannot be taken for granted. Accepting vaccine rejection without offering every potential vaccinee a clarifying and argumentative dialogue suggests ethical indifference to their fate. Recognising them as competent senders, recipients, and examiners of arguments, in contrast, may be the most appropriate understanding of patient-centered medicine and healthcare.

## Conclusion

As the title of this article suggests, we propose that the primacy of rationality should prevail over any claim to personal autonomy as freedom from interference. Personal decisions in healthcare should always be made prudently, and the greater their impact on the agent or third parties the more important the requirement of rationality. COVID-19 has shown that one cannot per se assume a given case of vaccine refusal to be the prima facie expression of a rational decision. However, the same applies to the state's requirement that citizens comply with a mandatory measure. The obligation to vaccinate is on shaky ground if valid objections on the part of the vaccinee are present. From here comes the need for dialogue, in which both sides inform each other, listen to the statements put forward, respond, and attempt to reconcile them in a plan of action. The purpose of this article is to demonstrate the constitutive role of argument herein: rather than threatening personal autonomy, it can be understood as a relational and dynamic process of claiming, refuting, and justifying validity to achieve clarity of will and responsibility in action.

**Abbreviations**
COVID-19: Coronavirus disease 2019
ECMO: Extracorporeal membrane oxygenation
mRNA: Messenger ribonucleic acid
SARS-CoV-2  : Severe acute respiratory syndrome coronavirus-2

## Consent for publication

All authors have approved the final manuscript and consented to publication.

## Notes

1 During the second wave of COVID-19 in Denmark and forth (Meier et al., 2023) (the third and fourth wave of COVID-19 from 15 March 2021 and after according to the European registry (Lorusso et al., 2021)), for patient characteristics see (Pedersen et al., 2025).

2 In 2021, vaccination reached approximately a coverage of 80 percent of the adult population (Meier et al., 2023).

3 At this time, the Danish Health Authority had not attributed any of the reported fatal events that occurred in the temporal context of vaccination of more than three million serum samples to the vaccine.

4 As the effectiveness and safety profile of vaccines differ, their social impact may not be fully comparable.

5 Only a minority of Danes comply fully with the official recommendations.

6 All means and services that serve to ward off, alleviate or cure diseases.

7 Such as wealth, age, gender, religion, political beliefs or ethnicity.

8 The primary distinction between the presented concept and the so-named risk-related standards for patients' decisional capacity–which require varying levels of cognitive competence based on the specific content of informed consent–is the constitutive role of argument (Meier et al., 2023).
